# A knotty problem: phytobezoar and small bowel occlusion as a complication of a gastro-jejunal catheter for continuous Duodopa infusion: a case report

**DOI:** 10.1093/jscr/rjac118

**Published:** 2022-03-26

**Authors:** Todor Ivanov, Ingrid Perlot, Laura Romero Stoca, Catherine Deleuze, Celine Rasmont, Jean Lemaitre

**Affiliations:** CHU Amboirse Paré, Department of Surgery, Mons, Belgique; CHU Ambroise Paré, Department of Gastro-Enterology, Mons, Belgique; CHU Amboirse Paré, Department of Surgery, Mons, Belgique; CHU Amboirse Paré, Department of Surgery, Mons, Belgique; CHU Amboirse Paré, Department of Surgery, Mons, Belgique; CHU Amboirse Paré, Department of Surgery, Mons, Belgique

## Abstract

We report a case of small bowel occlusion due to the formation of a bezoar around a knot at the distal end a gastro-jejunal catheter used for continuous levodopa/carbidopa intestinal gel (LCIG) in a patient with advanced Parkinson’s disease. The patient presented with a history of abdominal pain and vomiting starting 24 h before admission and frequent failure of his LCIG device for the past week. Small bowel occlusion along with a knot formation on the distal catheter was confirmed by contrast enhanced CT scan. After failure of endoscopic extraction, the patient was taken to theater. The presence of a knot and a bezoar was confirmed and extraction proceeded via transverse enterotomy without the need for bowel resection. Despite inhalation pneumonia and prolonged ileus, the patient recovered fully. LCIG treatment was reinstated a month later through new gastro-jejunal catheter. This case highlights a severe and surprising complication of LCIG treatment.

## INTRODUCTION

Advanced Parkinson’s disease has significant impacts on the health and quality of life of its sufferers [1]. A continuous infusion of carbidopa-levodopa intestinal gel (LCIG) is a treatment option for patients who present an unsatisfactory response to oral therapies. LCIG seems to be effective in the improvement of both motor and non-motor symptoms of PD as well as improving the quality of life and diminishing the caretaker burden [2]. The treatment is delivered via a jejunal catheter placed through a percutaneous endoscopic gastrostomy (PEG-J). The need for the long-term presence of an intestinal foreign body invariably leads to the possibility of complications. Adverse event rates are reported in the literature to be between 34 and 74% in regards to the presence of the PEG-J [3, 4]. The majority are mild to moderate and many can be resolved by replacing the PEG-J. Some, however, are severe and may require prompt surgical intervention. Intestinal perforations, mucosal ulcerations and intestinal occlusions have all been described [4, 5]. Such complications have the potential to lead to lasting morbidity in an already fragile patient population as well to the interruption of LCIG treatment with associated negative impacts on the quality of life. Guidelines and standardization regarding the monitoring of and the timing of replacement of the PEG-J do not yet exist. Here we describe the case of a 65-year-old male treated for intestinal occlusion due to the formation of a phytobezoar at the distal catheter tip.

## CASE REPORT

The patient is a 65-year-old man suffering from PD since 2007. His initial clinical course was marked by symptom aggravation on oral Levodopa therapy, presenting as severe dyskinesia and motor blockages during 25–50% of the day. His motor symptoms were combined with neuropsychological disturbances, including insomnia and severe depression as well as postural instability responsible for multiple falls. In this context, he was approved for LCIG treatment at the end of 2019 with good control of symptoms. His PEG-J was changed at the end of 2020 due to a blockage, with the discovery of ulcerative gastritis at the PEG-J site during endoscopy. Treatment was continued normally afterwards.

**Figure 1:**
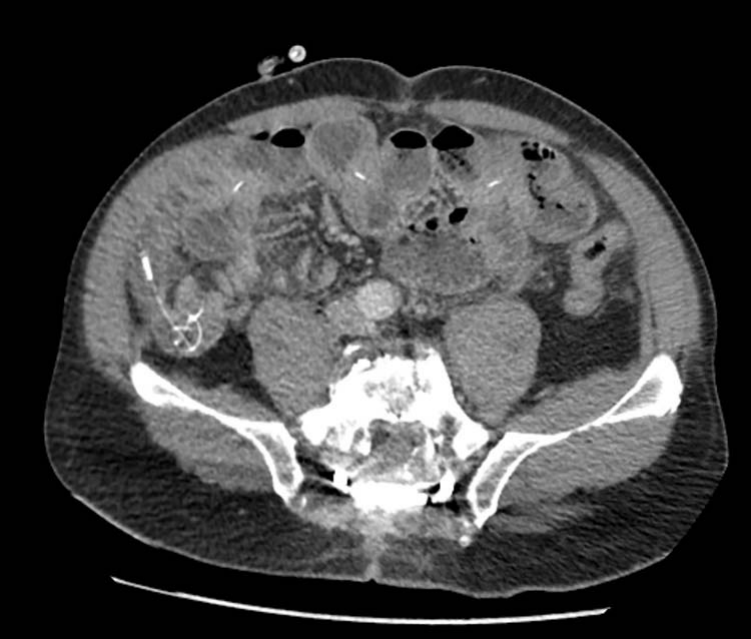
Preoperative CT scan showing intestinal occlusion and a knot formation.

**Figure 2:**
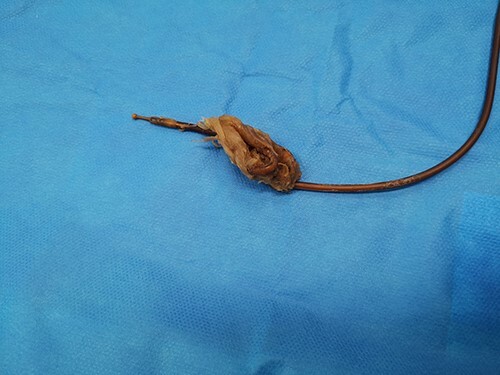
Extracted bezoar.

The patient presented to the emergency room for severe biliary vomiting for the past 24 h. He reported lack of gas and occlusion of his LCID pump for the past 48 h. His abdomen was distended and tender in the right flank, without guarding or signs of peritoneal irritation. Initial blood work showed an elevated white blood cell count at 13 880 10^3^/μl and elevated CRP at 140 mg/l. Initial CT scan showed an intestinal occlusion situation in the jejunum with major gastric distention (Fig. 1). The distal tip of the catheter was visualized at the occlusion site with the evident formation of a knot at its end. There were no signs of pneumoperitoneum or intestinal hypo-perfusion. A small quantity of free fluid was seen around the jejunum. A chest X-ray revealed a right lower lobe condensation, presumed to be inhalation pneumonia. A nasogastric tube was immediately placed, the patient was started on IV fluids and broad spectrum antibiotics and was hospitalized in the surgery department. Endoscopy was initially performed, which visualized mucosal ulcerations in the duodenum, and was ultimately unsuccessful in extracting the PEG-J due to firm adherence of the jejunal catheter. Emergency surgery was then performed via a midline laparotomy. The jejunum was markedly distended with subserosal ulcerations but was otherwise viable with good color and the presence of peristalsis. There was a small amount of clear free fluid. The jejunal catheter was extracted via an enterotomy, the formation of a knot was observed at its distal end and a large occlusive bezoar had formed around the knot (Fig. 2). The enterostomy was closed transversely with a running Vicryl 3-0 suture, an abdominal drain and the NG tube were left in place.

The immediate postoperative course was marked by prolonged ileus. The NG tube was removed at postoperative Day 5 and subsequent oral feeding was well tolerated. Peritoneal fluid microbiology was negative. Oral levodopa was reinstated via the NG tube on Day 2 with satisfactory control of neurological symptoms and the patient was transferred to neurology on Day 7 and then onto a rehabilitation facility. A new PEG-J was inserted a month after surgery and LCIG was able to be reintroduced without complications.

## DISCUSSION

This case is unusual in that phytobezoars more commonly form in the stomach and then migrate into the intestinal tract [6, 7]. The uncommon finding of a primary intestinal phytobezoar may possibly be due to the long-term presence of the jejunal catheter along with the formation of a distal knot, providing a platform for undigested fibers to congregate around. Moreover, this is not the first case of its kind reported in the literature in association with an LCIG catheter [8]. It would not be illogical to consider that since LCIG-treated patients maintain normal oral nutrition, they might be at an increased risk of such complications, especially if they consume foods with high fiber content [9]. Suggesting a low fiber diet might be a way to avoid such events. The importance of carefully monitoring the state of the jejunal catheter and having a high risk of suspicion for potential problems are recurrent conclusions in the current literature [4]. Cases of catheter kinking and blockage, duodenal pressure ulcers, intestinal abscesses, catheter migration and gastrostomy ring migration have all been reported [5, 10, 11]. There are as of yet no standardized protocols guiding the timing of tube replacements, which could potentially aid in preventing PEG-J-related complications in an already fragile population. Device complications are a known cause of LICG discontinuation [12] and avoiding them could help prolong effective treatment in certain patients.

## CONCLUSION

The long-term presence of an intestinal foreign body in LCIG patients may make them more susceptible to unusual complications as described above. Abdominal complaints and LCIG infusion dysfunction should prompt a rapid work up and consult with gastro-enterology and surgery to minimize morbidity and LCIG discontinuation in this patient population.
